# Comparison of Activation Patterns in Mirror Neurons and the Swallowing Network During Action Observation and Execution: A Task-Based fMRI Study

**DOI:** 10.3389/fnins.2020.00867

**Published:** 2020-08-21

**Authors:** Ying-hua Jing, Tuo Lin, Wan-qi Li, Cheng Wu, Xue Li, Qian Ding, Man-feng Wu, Guang-qing Xu, Yue Lan

**Affiliations:** ^1^Department of Rehabilitation Medicine, Guangzhou First People’s Hospital, School of Medicine, South China University of Technology, Guangzhou, China; ^2^Department of Rehabilitation Medicine, Guangzhou First People’s Hospital, Guangzhou Medical University, Guangzhou, China; ^3^Department of Rehabilitation Medicine, Beijing Tian Tan Hospital, Capital Medical University, Beijing, China; ^4^China National Clinical Research Center for Neurological Diseases, Beijing, China

**Keywords:** mirror neurons, swallowing network, action observation, action execution, functional magnetic resonance imaging

## Abstract

**Background:**

Observation of a goal-directed motor action can excite the respective mirror neurons, and this is the theoretical basis for action observation (AO) as a novel tool for functional recovery during stroke rehabilitation. To explore the therapeutic potential of AO for dysphagia, we conducted a task-based functional magnetic resonance imaging (fMRI) study to identify the brain areas activated during observation and execution of swallowing in healthy participants.

**Methods:**

Twenty-nine healthy volunteers viewed the following stimuli during fMRI scanning: an action-video of swallowing (condition 1, defined as AO), a neutral image with a Chinese word for “watching” (condition 2), and a neutral image with a Chinese word for “swallowing” (condition 3). Action execution (AE) was defined as condition 3 minus condition 2. One-sample *t*-tests were performed to define the brain regions activated during AO and AE.

**Results:**

Many brain regions were activated during AO, including the middle temporal gyrus, inferior frontal gyrus, pre- and postcentral gyrus, supplementary motor area, hippocampus, brainstem, and pons. AE resulted in activation of motor areas as well as other brain areas, including the inferior parietal lobule, vermis, middle frontal gyrus, and middle temporal gyrus. Two brain areas, BA6 and BA21, were activated with both AO and AE.

**Conclusion:**

The left supplementary motor area (BA6) and left middle temporal gyrus (BA21), which contains mirror neurons, were activated in both AO and AE of swallowing. In this study, AO activated mirror neurons and the swallowing network in healthy participants, supporting its potential value in the treatment of dysphagia.

## Introduction

Mirror neurons are a specific neuronal population that discharge not only during the performance of a particular action, but also during observation of others performing a similar action (Cook et al., 2014; Marshall, 2014). These neurons are found throughout the brain, including in the Broca’s area, ventral premotor region, premotor cortex (PMC), posterior regions of the inferior frontal gyrus (IFG), inferior parietal lobule (IPL), insula, amygdala, and prefrontal cortex (PFC) (Casile et al., 2011; Kilner and Lemon, 2013; Cook et al., 2014; Zhang et al., 2018). Current theories in cognitive neuroscience suggest that mirror neurons are central to the basic neural mechanisms underlying action recognition, motion intention coding, and motor learning (Maranesi et al., 2014; Amoruso et al., 2018; Catmur et al., 2018; Mazurek et al., 2018). Previous studies revealed a neuronal mechanism of mirror neurons that matches perception and action as well as cognition and motility, and permits action recognition and understanding (Rizzolatti and Sinigaglia, 2016; Tramacere et al., 2017). In other words, observation of others’ actions can activate brain regions similar to those involved in the execution of that action and then can induce the same action. For example, Fawcett et al. illustrated that infants learn to replicate others’ joint activity through observation (Fawcett and Liszkowski, 2012). Given the properties of the action observation–execution matching mechanism, mirror neurons may influence plasticity-related functional reorganization after stroke or brain injury, re-activating the top-down neural pathway, leading to recovery of motor function (Sale and Franceschini, 2012; Small et al., 2013). In recent years, action observation (AO), in which patients observe a specific video describing an action to recruit the relevant action-related mirror neurons and enable a direct matching between others’ gestures and their own motor system, has emerged as a novel and effective option as an add-on intervention to rehabilitation therapy (Hetu et al., 2010; Sale and Mattingley, 2013; Bassolino et al., 2014; Vesia et al., 2019). To date, this approach has been successfully applied in the rehabilitation of upper limb motor functions in chronic stroke patients (Borges et al., 2018), in the language rehabilitation of post-stroke aphasia patients (Gili et al., 2017), in motor recovery of Parkinson’s disease patients (Pelosin et al., 2013), including those presenting with freezing of gait (Pelosin et al., 2018), and in children with cerebral palsy (Kirkpatrick et al., 2016).

Swallowing is a coordinated physiological process, involving complex neuromuscular interactions (Ertekin and Aydogdu, 2003). Extensive research has shown that the swallowing network includes the brain regions of the primary sensorimotor system, supplementary motor area (SMA), insula, cingulate cortex, IFG, IPL, temporal lobe, precuneus, cerebellum, and brain stem (Hamdy et al., 1999a; Lang, 2009; Soros et al., 2009; Lima et al., 2015; Toogood et al., 2017). When any area of the swallowing network is damaged, dysphagia occurs (Domenech and Kelly, 1999; Gonzalez-Fernandez and Daniels, 2008). Dysphagia can result in serious complications, including pneumonia, malnutrition, dehydration, and even death, and is strongly linked to a deteriorated quality of life (Clavé and Shaker, 2015; Rommel and Hamdy, 2016). Regrettably, because traditional dysphagia therapies (e.g., compensatory strategies, external stimulation of oral, and pharyngeal structures) are aimed at recovering the function of swallowing biomechanics rather than directly repairing the central nervous system (Martino and McCulloch, 2016; Easterling, 2017; Lazarus, 2017), the cycle of rehabilitation is long and the outcome of clinical treatment is typically poor. Therefore, repairing the damaged networks directly to improve swallowing function has become a challenge demanding a prompt answer. We found that some brain areas of swallowing regulation overlap those with mirror neurons, such as the motor regions, IFG, IPL and others. Additionally, animal studies have shown that observation of eating behavior can stimulate observers to execute swallowing-related action (Rizzolatti and Craighero, 2004). Based on the aforementioned exploration of the literature, we speculated that AO might be useful in treating dysphagia. In contrast to other body movements, swallowing is regulated by motor and sensory feedback and is particularly difficult to observe directly (Dodds, 1989). As yet, there is paucity of evidence demonstrating that an action-video of swallowing can activate mirror neurons and the swallowing network, prompting us to conduct the current experiment.

Functional magnetic resonance imaging (fMRI), which detects changes in the blood oxygen level-dependent (BOLD) signal as an indirect measure of neuronal activity, is a powerful tool for exploring brain functions non-invasively (Ogawa et al., 1992). Compared to other neuroimaging methods [e.g., electroencephalography (EEG), magnetoencephalography (MEG), near-infrared spectroscopy (NIRS)], fMRI offers good contrast resolution and excellent spatial resolution for accurately determining the location and activation patterns of a neural source. The rapid development of neuroimaging technology has facilitated task-based fMRI, which can accurately reveal the brain regions that are activated when one executes a certain task. This approach provides a good foundation for our research on the activation of mirror neurons and the swallowing network induced by AO (Ugurbil, 2016). In 12 healthy adults, Kawai et al. (2009) used fMRI to verify that visual and audiovisual stimuli related to swallowing motion contribute to activation of the brain regions related to swallowing. However, in comparison to a still image or auditory stimuli associated with swallowing, action-video stimuli of swallowing promote better integral transmission of coherent information regarding the swallowing motion. Additionally, the single-trial paradigm used in Kawai et al.’s study can track only the chronological changes activated by a single stimulus, decreases the BOLD-signals caused by the stimulus, and offers weak contrast-to-noise ratios. Moreover, previous studies were limited by small sample sizes. To address these problems and improve the detection efficiency for mirror neurons, we devised a block-design fMRI study in 29 health volunteers. This type of design was previously shown to be applicable for the swallowing task by Soltysik and Hyde (2006).

In the current study, based on our assumption that AO of swallowing in daily life can activate brain regions similar to those required for the action execution (AE) of swallowing, we employed task-based fMRI in a reasonably large subject population to identify the regions of the brain that are activated during observation of the action-video of swallowing and the neuronal responses that occur during swallowing, as well as to investigate interactions among the brain areas activated by the task. Furthermore, we examined whether the specific swallowing video can modulate voluntarily participants’ mirror neurons to induce brain activity related to swallowing and estimated whether AO represents a feasible intervention for dysphasia that warrants further research in patients.

## Materials and Methods

### Participants

Twenty-nine healthy volunteers (15 female, 14 male, mean age: 22.76 ± 2.63 years; education level: 15.48 ± 1.06 years) participated in the study after providing written informed consent. All participants had normal or corrected-to-normal vision, and volunteers were excluded if they had a personal history of dysphagia, current sore throat due to respiratory infection or chronic pharyngitis, neurological or psychiatric illness, or drug or alcohol abuse. The experiment was approved by the Medical Ethics Committee of medical college of South China University of Technology and was performed in accordance with the ethical standards of the Declaration of Helsinki.

### Experimental Design

Before the fMRI experiment, all participants underwent an operant training to ensure they fully understand the entire procedure, including the task-related functional and structural scans.

**FIGURE 1:**
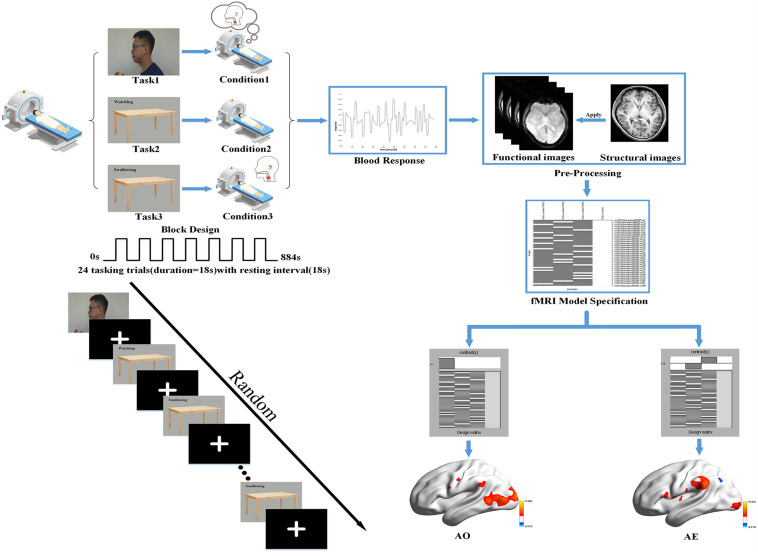
Basic experimental scheme. We used a block design for the task performed in our fMRI experiment with a randomized task sequence. The steps of the first-level analysis are presented using the data for one participant as an example, and the BrainNetViewer (https://www.nitrc.org/projects/bnv/) was used to illustrate the brain activation maps.

During the functional image acquisition, participants were asked to lie down quietly in a comfortable supine position on the magnetic resonance scanner. They then viewed the session through a tilted mirror mounted above their eyes on the head coil, and completed a total session lasting 14.4 min and comprised of three different task blocks of 36 s (with 18 s trial duration and 18 s rest period). In the task blocks, three stimuli were presented: a video of the left profile of a man biting into an apple, chewing, and then swallowing; a neutral picture of a desk with a Chinese word for “watching”; and the same neutral picture with a Chinese word for “swallowing.” A dark image was shown during the rest periods. When the video of swallowing appeared on the screen, participants were instructed to attentively watch and understand the swallowing movements of the man. When the image of the desk with the “swallowing” message was shown, they were instructed to execute the swallowing motion in a regular, comfortable, self-paced rhythm. When viewing the image with the word “watching,” they needed to remain quiet (with the absence of swallowing movements or thought) and focus on the screen. Each block was repeated eight times in pseudo-random order with sequence randomized according to a computer-generated list of random numbers. A 10 s blank block was allowed for elimination of the influence of blood signal saturation and adaption to the MRI environment initially. In the same way, a 10 s blank block was used to avoid end-effects and data corruption at the end of the session. Then, a T1 scan for the structural image was performed after the task session, in which participants were instructed to remain awake but relaxed with their eyes closed, remaining motionless as much as possible. The detailed experimental design is illustrated in Figure 1.

### MRI Data Acquisition

All brain imaging data were acquired on a 3.0 T MRI scanner (Siemens, Erlangen, Germany) with an 8-channel head coil at Guangzhou First People’s Hospital, China. The MRI scanning sessions consisted of an approximately 14.4 min task-based session and a 9 min anatomical scan. All participants underwent T2-sensitive gradient reflection echo (GRE) sequence scanning (parameters: TR = 2000 ms, TE = 21 ms, flip angle = 78°, matrix = 64 × 64, slice thickness = 4.0 mm, voxel dimensions = 3.5 × 3.5 × 4.0 mm), which provided whole-brain coverage in 33 slices during task completion. T1-weighted structural images were recorded using a 3D magnetization-prepared rapid gradient echo (MPRAGE) sequence (parameters: TR = 2530 ms, TE = 2.93 ms, TI = 900 ms, flip angle = 9°, matrix = 256 × 256, slice thickness = 1.0 mm, voxel dimensions = 1.0 × 1.0 × 1.0 mm). The MRI sequence and image-acquisition parameters were kept constant during the scanning period. Participants wore earplugs to reduce scanner noise, and pads were used to reduce head motion. During the MRI scanning sessions, participants lay supine on the scanner bed and viewed the experimental tasks on a screen through a mirror mounted onto the head coil.

### fMRI Data Preprocessing

Image preprocessing was performed using RESTplus (Jia et al., 2019), which runs with the statistical parametric mapping software SPM12^1^. Briefly, all images were preprocessed with the following steps:

I. Conversion of the DICOM data format into NIFTI format and visual inspection for quality for the convenience of data processing.
II. Slice timing to correct for differences in the time of slice acquisition.
III. Head motion correction using six parameters, with the first volume as a reference slice.
IV. Normalization of all functional images to the Montreal Neurological Institute (MNI) space via the Rigid Body deformation fields derived from tissue segmentation of structural images (resampling voxel size = 3 mm × 3 mm × 3 mm) for accurate spatial localization within the brain.
V. Spatial smoothing after normalization to improve the quality of group-level statistics with an isotropic Gaussian kernel with a full width at half maximum (FWHM) of 6 mm.

No participants were excluded from further analysis due to image registration error or large head motion (more than 3.0 mm of maximal translation in any direction of x, y or z or 3.0° of maximal rotation throughout the course of scanning).

### fMRI Data Analysis

SPM12 was used in a block design to analyze functional whole-brain data with the purpose of determining group effects. The General Linear Model (GLM) was used in the first-level analysis. First, to observe the activation in each condition, we used the following design matrix: video task (condition 1) versus rest period; neutral picture of a desk with a Chinese word for “watching” (condition 2) versus rest period; neutral picture with a Chinese word for “swallowing” (condition 3) versus rest period. Second, whole-brain analysis of the following contrasts of task conditions was performed: we defined condition 1 as the AO and condition 3 minus condition 2 as the action execution (AE). Hence, the analysis revealed the activation patterns of mirror neurons during AO and the activation patterns of the swallowing network during AE. In order to detail and check the activation pattern in each participant, the resultant T-maps were considered with a threshold of *P* < 0.001 uncorrected for multiple comparisons. The first-level analysis is outlined in Figure 1.

In the second-level analysis, the statistical analysis, the beta weights of two contrasts were statistically compared separately using one sample *t*-test, with the age and sex of participants as covariates. The results were corrected for multiple comparisons using the false discovery rate (FDR) correction at the voxel-level, and the significance threshold was set at FDR-corrected *P* < 0.01 with a minimum cluster size (the number of voxels) of 10 voxels.

## Results

### AO-Condition 1

**FIGURE 2:**
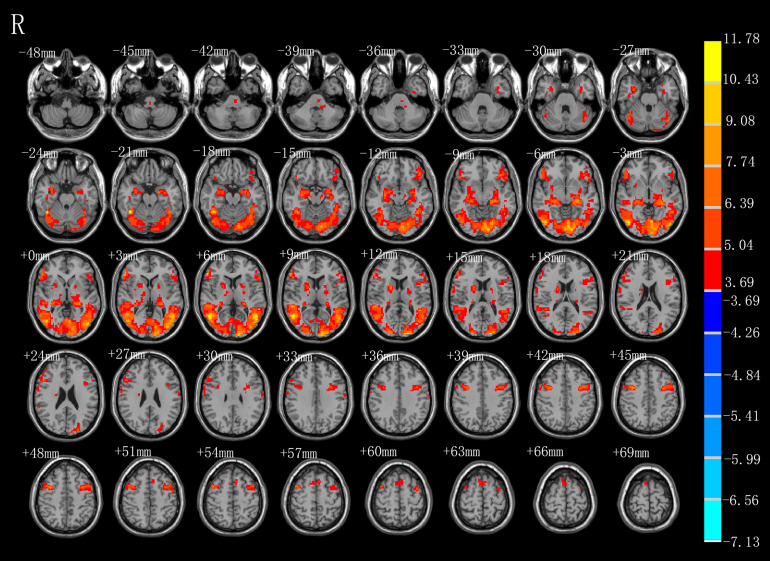
T-score map showing activation for AO (hot colors) in transverse slices (*P* < 0.01, FDR corrected, cluster size > 10 voxels).

As shown in Figure 2 and Table 1, observation of the action-video of swallowing motion led to activation patterns in the bilateral middle temporal gyrus (BA37\21), left pre- and postcentral gyrus (BA6\4), left hippocampus, right inferior frontal gyrus (BA45), right SMA (BA6), left brainstem, and pons (*P* < 0.01, FDR corrected, cluster size > 10 voxels).

**TABLE 1:** Brain regions activated by AO.

Regions (AAL)	BA	Cluster size	Peak *T*-value	MNI coordinate (mm)		
				*X*	*Y*	*Z*
**AO**						
Temporal_Mid_R	37	5270	11.7835	51	−57	6
Frontal_Inf_Tri_R	45	325	10.1845	57	36	6
Precentral_R	6	272	9.6466	36	3	45
Supp_Motor_Area_R	6	109	5.5840	9	12	72
Precentral_L	6	688	7.3714	−36	6	45
Hippocampus_L	N.A	589	8.8161	−24	−24	−6
Postcentral_L	4	16	4.6161	−63	−9	30
Temporal_Mid_L	21	10	4.2366	−57	−24	−6
Left Brainstem	N.A	17	4.7916	−9	−21	−42
Pons	N.A	10	4.6971	−18	−33	−39

*BA, Brodman’s area; Cluster size, the number of voxels; MNI, Montreal Neurological Institute; R right, L left; P < 0.01, FDR corrected, cluster size > 10 voxels.*

### AE-Condition 3 Minus Condition 2

**FIGURE 3:**
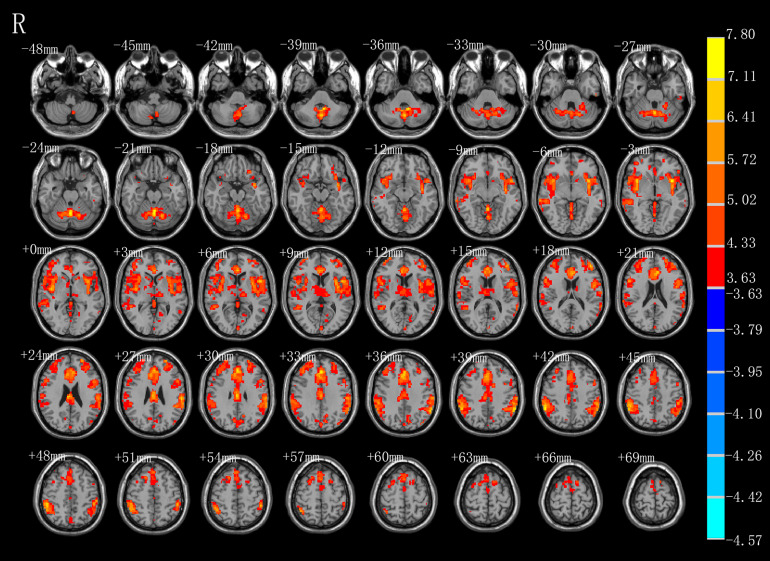
T-score map showing stronger activation of brain areas with AE (hot colors) in transverse slices (*P* < 0.01, FDR corrected, cluster size > 10 voxels).

As shown in Figure 3 and Table 2, compared with the task image without action, execution of swallowing movements activated the brain network in the motor areas (left SMA [BA6]), bilateral inferior parietal lobule (BA40), left anterior cingulate (BA32), vermis of the cerebellum, left middle frontal gyrus (BA45), left superior frontal gyrus (BA11), left thalamus, left precuneus (BA7), left cuneus, left middle temporal gyrus (BA37\21), left inferior temporal gyrus (BA20), left middle occipital gyrus (BA39), left calcarine (BA18), and bilateral caudate (*P* < 0.01, FDR corrected, cluster size > 10 voxels).

**TABLE 2:** Brain regions activated more strongly with AE.

Regions (AAL)	BA	Cluster size	Peak *T*-value	MNI coordinate (mm)		
				*X*	*Y*	*Z*
**AE**						
Parietal_Inf_R	40	2728	7.4847	57	−48	42
Caudate_R	N.A	17	4.9357	15	21	3
Parietal_Inf_L	40	1995	7.5035	−60	−48	39
Cingulum_Ant_L	32	1829	7.2223	0	36	30
Frontal_Mid_L	45	415	6.6479	−42	45	15
Thalamus_L	N.A	293	4.6826	−15	−18	6
Cuneus_L	N.A	150	5.2192	−9	−75	36
Supp_Motor_Area_L	6	56	4.8467	−12	12	66
Calcarine_L	18	39	4.2421	−12	−75	9
Caudate_L	N.A	32	5.2077	−15	18	0
Occipital_Mid_L	39	27	5.2851	−42	−78	36
Precuneus_L	7	24	4.1871	0	−72	48
Temporal_Mid_L	37	16	4.3757	−60	−57	3
Frontal_Sup_Orb_L	11	13	4.5128	−21	57	−3
Temporal_Mid_L	37	11	4.4436	−60	−57	15
Temporal_Mid_L	21	11	4.1524	−66	−39	−6
Temporal_Inf_L	20	10	5.0509	−54	−18	−30
Vermis_6	N.A	1230	7.8017	0	−60	−24

*BA, Brodman’s area; Cluster size, the number of voxels; MNI, Montreal Neurological Institute; R right, L left; P < 0.01, FDR corrected, cluster size > 10 voxels.*

### AO Versus AE

**FIGURE 4:**
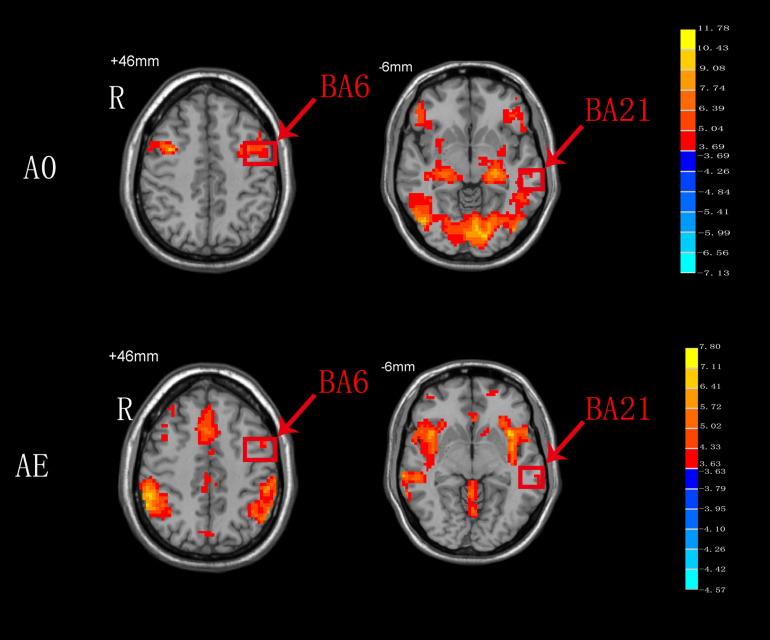
T-score map showing the brain areas activated during both AO and AE (hot colors) in transverse slices (*P* < 0.01, FDR corrected, cluster size > 10 voxels).

As shown in Figure 4, BA6 and BA21 were activated during both AO and AE states.

## Discussion

In the present study, we examined and compared the patterns of brain activation in healthy participants as they observed an action-video and as they actually executed the swallowing motion using task-based fMRI. We identified the brain regions of BA6 and BA21 as commonly activated during the two sessions and speculated on the role of AO in swallowing.

### AO-Condition 1

During condition 1, observation of the video of the task, the most critical task of our experiment, we observed activated of not only similar mirror neurons reported in a prior study (Cook et al., 2014), but also increased activity of classical swallowing network. Consistent with the findings in the previous review (Ferrari et al., 2017), activation of brain regions in the left pre- and postcentral gyrus (BA6\4), right inferior frontal gyrus (BA45), and right supplementary motor area (BA6) did correspond to mouth mirroring in the present study. In our results, the bilateral precentral (BA6) and right SMA (BA6) were strongly activated when the healthy participants watched the video of swallowing action, which is consistent with the findings of a previous MEG study (Ushioda et al., 2012). In general, the SMA (BA6), located in the superior and middle frontal gyri, often processes sequential motions, especially those associated with the beginning of the motions, and the precentral gyrus (BA6), including the premotor areas and primary motor cortex, is involved in complex experienced motions. However, different from the prior MEG study, observation of our action-video of swallowing led to the activation of many other brain regions (e.g., BA37, BA45, BA4, and BA21) rather than BA40. In addition to the intrinsic advantage of fMRI for extracting deep or multiple BOLD signals, we considered that observation of the action-video for a daily performance activity may be better than animation or auditory stimuli for activating the brain regions of mirror neurons related to swallowing. Specifically, considering our results in comparison with those of prior studies, due to the complementary role of the inferior frontal gyrus (BA45) in swallowing regulation, it is possible that activation of BA45 during our video task is associated with taste and imagery of the apple in the action-video. In addition, the left brainstem and pons are associated with voluntary swallowing more than spontaneous swallowing in humans, which might explain why observation of the action-video of swallowing proactively stimulated swallowing among healthy participants (Ertekin, 2011). The hippocampus is the site in the brain responsible for memory and learning and may therefore be conducive to evoked memory of swallowing and play a role in the swallowing process (Downar et al., 2000). We also hypothesized that the hippocampus receives information from the action-video, coordinates with the precentral gyrus, and then relays the neural signals to SMA, and further research is needed to confirm this hypothesis. More importantly, the most meaningful finding from this experiment is that activation of the specific brain regions, including the bilateral middle temporal gyrus (BA37\21), inferior frontal gyrus (BA45), primary motor cortex, and SMA (BA6), is consistent with the hypothesis of Small et al. that the mirror neurons activated by the action-video of swallowing may promote reorganization of the cortical motor loop and thereby improve swallowing function (Small et al., 2012).

### AE-Condition 3 Minus Condition 2

In line with the previous studies, our comparison of condition 2 with condition 3, which excluded the visual effects and emotional reactions, showed that the AE of swallowing activated many different brain areas related to swallowing. Martin et al. reported that the SMA (BA6) and anterior cingulate (BA32) are vital for the control of voluntary swallowing (Martin et al., 2001). As mentioned above, the SMA (BA6) plays a dynamic role in the execution of different swallowing tasks and is mainly involved in comprehensive oral movements. One explanation for the activation of the anterior cingulate (BA32) is that voluntary saliva swallowing may rely on attention and concentration. Additionally, Kawai et al. reported that the pre-SMA receives input signals from the cingulate gyrus and pre-frontal area to facilitate the performance of a series of swallowing motions (Kawai et al., 2009). During the execution of swallowing, we propose that activation of the left superior frontal gyrus (BA11) and left middle frontal gyrus (BA45), which are associated with emotion, motivation, and learning-set formation, reflects the processing of swallowing information and its delivery to the SMA. The temporal gyrus that was activated in prior experiments and corresponds to the left middle temporal gyrus (BA37\21) and left inferior temporal gyrus (BA20) in our experiment, has been implicated in taste and imagery of food and may play a supplementary role in the regulation of swallowing and feeding (Hamdy et al., 1999b; Ertekin and Aydogdu, 2003). However, it is also possible that the noise produced by the MRI scanner and the sounds of swallowing by themselves activated the auditory cortex. Because all participants wore ear plugs, we instead suggest that the activation of the temporal gyrus may reflect the acoustic correlates of swallowing. In this study, activation of bilateral inferior parietal lobules (BA40) located near the insula and bilateral caudate, which are related to oral movements and sensation, may be a characteristic of bilateral movements such as swallowing, as reported by a previous MEG study (Ushioda et al., 2012). However, while the meta-analysis by Soros et al. observed activation in the basal ganglia, they also reported involvement of the putamen but not of the caudate during swallowing, in contrast with the present findings (Soros et al., 2009). Moreover, the cluster of precuneus (BA7), cuneus, and calcarine (BA18) also are likely to play a sensory role in the control of swallowing (Kober et al., 2019). Activation of the vermis of the cerebellum and thalamus was shown to contribute to swallowing and to effective connectivity within the swallowing network in recent neuroimaging studies (Ushioda et al., 2012; Babaei et al., 2013). The brain region of the middle occipital gyrus (BA39) is a well-established component of the visual cortex that processes visual information. We speculate that, due to the difference in the Chinese words presented in condition 2 versus condition 3, the activity in the left middle occipital gyrus (BA39) mainly aims to distinguish different task instructions. Yet, Hamdy et al. proposed that the left brain is the dominant side for the swallowing network (Hamdy et al., 1999a), a conclusion that is not supported by our findings. Indeed, in our study (Figure 1), large clusters of activation were observed in the right brain whereas the activated areas in the left brain were dispersed among a number of spatially discrete cortical regions. Further research is required to determine the dominant hemisphere for swallowing.

### AO Versus AE

In the present study, both the BA6 and BA21 areas were activated during both AO and AE. The Ba6 brain area, which includes the premotor cortex and SMA, is a dominant area for motor function bilaterally. Undoubtedly, BA6 responds consistently during AE of swallowing, and this findings is in agreement with the results of a meta-analysis on swallowing (Soros et al., 2009). Compared to AE of swallowing, AO of swallowing also activated BA6, and this might be related to the activation of mirror neurons in this area. We hypothesize that activation of BA6 appears is not only motor marker, but also a marker of the ability of mirror neurons to relay visual information to the executive control network to achieve goal-directed behaviors (Zhang et al., 2018). Although the BA21 brain area was also activated during both AO and AE, the specific anatomical location of this activation remains unclear (Figure 4). We hypothesize that BA21 actively focuses on visual processing during AO (Small et al., 2012), whereas it may participate in swallowing regulation with taste and imagery of food during AE (Ertekin and Aydogdu, 2003). The detailed activation pattern requires further exploration.

### Study Limitations and Future Directions

The present study provided a simple comparison of neuronal correlates between mirror neurons and the swallowing network, and showed that the left supplementary motor area (BA6) and left middle temporal gyrus (BA21) may be foundational to the role of AO in treatment for dysphagia. However, our results did not reveal the precise neural mechanisms of the two neuronal networks, and thus, we are planning future neuroimaging analyses to elucidate these mechanisms. Furthermore, a limitation of the present study is that we could not control for head movement during swallowing. Because swallowing and head movement are highly correlated, we did not use head motion as a covariate in our statistical analysis to rule out the effects of the head motion on the current results.

Dysphagia seriously affects the general condition as well as the quality of life of the patient. Novel treatments to repair the injured nerves are urgently needed to improve the clinical efficacy. Our findings suggest that AO can be applied to dysphagia directly via impacting the connection of mirror neurons and swallowing network. Additionally, we can stimulate BA6 and BA21 to enhance and regulate the swallowing network and mirror neurons dynamically via non-invasive stimulation, and then repair the swallowing network by AO. Further studies in dysphagia patients are warranted to evaluate whether the action-video of swallowing could be used as a stimulus for AO in the treatment of dysphagia and to elucidate the neural mechanisms of AO.

## Conclusion

Our results preliminarily indicate that AO using an action-video of swallowing practically activates the mirror neurons related to swallowing in healthy individuals. We believe that AO can be used as an effective therapy to repair the damaged swallowing network for dysphagia. Particular attention should be paid to two brain regions, BA6 and BA21, which were commonly activated during the observation and execution of swallowing, in future patient studies.

## Data Availability Statement

The raw data supporting the conclusions of this article will be made available by the authors, without undue reservation.

## Ethics Statement

The studies involving human participants were reviewed and approved by the Medical Ethics Committee of medical college of South China University of Technology. The patients/participants provided their written informed consent to participate in this study. Written informed consent was obtained from the individual(s) for the publication of any potentially identifiable images or data included in this article.

## Author Contributions

YL and GX guided the experiment design. YJ and TL conceived, designed, and completed the experiment. YJ analyzed the data and drafted the manuscript. TL reviewed and edited the manuscript. CW, XL, QD, and MW collected the data. All authors contributed to the article and approved the submitted version.

## Conflict of Interest

The authors declare that the research was conducted in the absence of any commercial or financial relationships that could be construed as a potential conflict of interest.

## Footnotes

1 http://www.fil.ion.ucl.ac.uk/spm

## References

[B1] Amoruso L. Finisguerra A. Urgesi C. (2018). Contextualizing action observation in the predictive brain: causal contributions of prefrontal and middle temporal areas. *Neuroimage* 177 68–78. 10.1016/j.neuroimage.2018.05.020 29753844

[B2] Babaei A. Ward B. D. Siwiec R. M. Ahmad S. Kern M. Nencka A. (2013). Functional connectivity of the cortical swallowing network in humans. *NeuroImage* 76 33–44. 10.1016/j.neuroimage.2013.01.037 23416253 PMC4130480

[B3] Bassolino M. Campanella M. Bove M. Pozzo T. Fadiga L. (2014). Training the motor cortex by observing the actions of others during immobilization. *Cereb. Cortex* 24 3268–3276. 10.1093/cercor/bht190 23897648 PMC4224244

[B4] Borges L. R. Fernandes A. B. Melo L. P. Guerra R. O. Campos T. F. (2018). Action observation for upper limb rehabilitation after stroke. *Cochrane Database Syst. Rev.* 10:CD011887. 10.1002/14651858.CD011887.pub2 30380586 PMC6517007

[B5] Casile A. Caggiano V. Ferrari P. F. (2011). The mirror neuron system: a fresh view. *Neuroscientist* 17 524–538. 10.1177/1073858410392239 21467305 PMC3743423

[B6] Catmur C. Thompson E. L. Bairaktari O. Lind F. Bird G. (2018). Sensorimotor training alters action understanding. *Cognition* 171 10–14. 10.1016/j.cognition.2017.10.024 29102804

[B7] Clavé P. Shaker R. (2015). Dysphagia: current reality and scope of the problem. *Nat. Rev. Gastroenterol. Hepatol.* 12 259–270. 10.1038/nrgastro.2015.49 25850008

[B8] Cook R. Bird G. Catmur C. Press C. Heyes C. (2014). Mirror neurons: from origin to function. *Behav. Brain Sci.* 37 177–192. 10.1017/S0140525X13000903 24775147

[B9] Dodds W. J. (1989). Physiology of swallowing. *Dysphagia* 3 171–178. 10.1007/bf02407219 2700955

[B10] Domenech E. Kelly J. (1999). Swallowing disorders. *Med. Clin. North Am.* 83 97–113. 10.1016/s0025-7125(05)70090-0 9927963

[B11] Downar J. Crawley A. P. Mikulis D. J. Davis K. D. (2000). A multimodal cortical network for the detection of changes in the sensory environment. *Nat. Neurosci.* 3 277–283. 10.1038/72991 10700261

[B12] Easterling C. (2017). 25 years of dysphagia rehabilitation: what have we done, what are we doing, and where are we going? *Dysphagia* 32 50–54. 10.1007/s00455-016-9769-8 28044204

[B13] Ertekin C. (2011). Voluntary versus spontaneous swallowing in man. *Dysphagia* 26 183–192. 10.1007/s00455-010-9319-8 21161279

[B14] Ertekin C. Aydogdu I. (2003). Neurophysiology of swallowing. *Clin. Neurophysiol.* 114 2226–2244. 10.1016/s1388-2457(03)00237-2 14652082

[B15] Fawcett C. Liszkowski U. (2012). Observation and initiation of joint action in infants. *Child Dev.* 83 434–441. 10.1111/j.1467-8624.2011.01717.x 22277061

[B16] Ferrari P. F. Gerbella M. Coude G. Rozzi S. (2017). Two different mirror neuron networks: the sensorimotor (hand) and limbic (face) pathways. *Neuroscience* 358 300–315. 10.1016/j.neuroscience.2017.06.052 28687313 PMC6063080

[B17] Gili T. Fiori V. De Pasquale G. Sabatini U. Caltagirone C. Marangolo P. (2017). Right sensory-motor functional networks subserve action observation therapy in aphasia. *Brain Imaging Behav.* 11 1397–1411. 10.1007/s11682-016-9635-1 27734301

[B18] Gonzalez-Fernandez M. Daniels S. K. (2008). Dysphagia in stroke and neurologic disease. *Phys. Med. Rehabil. Clin. N. Am.* 19 867–888. 10.1016/j.pmr.2008.07.001 18940646

[B19] Hamdy S. Mikulis D. J. Crawley A. Xue S. Lau H. Henry S. (1999a). Cortical activation during human volitional swallowing: an event-related fMRI study. *Am. J. Physiol.* 277 G219–G225. 10.1152/ajpgi.1999.277.1.G219 10409170

[B20] Hamdy S. Rothwell J. C. Brooks D. J. Bailey D. Aziz Q. Thompson D. G. (1999b). Identification of the cerebral loci processing human swallowing with H2(15)O PET activation. *J. Neurophysiol.* 81 1917–1926. 10.1152/jn.1999.81.4.1917 10200226

[B21] Hetu S. Gagne M. Jackson P. L. Mercier C. (2010). Variability in the effector-specific pattern of motor facilitation during the observation of everyday actions: implications for the clinical use of action observation. *Neuroscience* 170 589–598. 10.1016/j.neuroscience.2010.07.015 20633609

[B22] Jia X.-Z. Wang J. Sun H.-Y. Zhang H. Liao W. Wang Z. (2019). RESTplus: an improved toolkit for resting-state functional magnetic resonance imaging data processing. *Sci. Bull.* 64 953–954. 10.1016/j.scib.2019.05.008 36659803

[B23] Kawai T. Watanabe Y. Tonogi M. Yamane G. Y. Abe S. Yamada Y. (2009). Visual and auditory stimuli associated with swallowing: an FMRI study. *Bull. Tokyo Dent. Coll.* 50 169–181. 10.2209/tdcpublication.50.169 20179392

[B24] Kilner J. M. Lemon R. N. (2013). What we know currently about mirror neurons. *Curr. Biol.* 23 R1057–R1062. 10.1016/j.cub.2013.10.051 24309286 PMC3898692

[B25] Kirkpatrick E. Pearse J. James P. Basu A. (2016). Effect of parent-delivered action observation therapy on upper limb function in unilateral cerebral palsy: a randomized controlled trial. *Dev. Med. Child Neurol.* 58 1049–1056. 10.1111/dmcn.13109 27038153

[B26] Kober S. E. Grössinger D. Wood G. (2019). Effects of motor imagery and visual neurofeedback on activation in the swallowing network: a real-time fMRI study. *Dysphagia* 34 879–895. 10.1007/s00455-019-09985-w 30771088 PMC6825652

[B27] Lang I. M. (2009). Brain stem control of the phases of swallowing. *Dysphagia* 24 333–348. 10.1007/s00455-009-9211-6 19399555

[B28] Lazarus C. L. (2017). History of the use and impact of compensatory strategies in management of swallowing disorders. *Dysphagia* 32 3–10. 10.1007/s00455-016-9779-6 28130600

[B29] Lima M. S. Mangilli L. D. Sassi F. C. Andrade C. R. (2015). Functional magnetic resonance and swallowing: critical literature review. *Braz. J. Otorhinolaryngol.* 81 671–680. 10.1016/j.bjorl.2015.08.006 26394917 PMC9442730

[B30] Maranesi M. Livi A. Fogassi L. Rizzolatti G. Bonini L. (2014). Mirror neuron activation prior to action observation in a predictable context. *J. Neurosci.* 34 14827–14832. 10.1523/jneurosci.2705-14.2014 25378150 PMC6608372

[B31] Marshall J. (2014). Mirror neurons. *Proc. Natl. Acad. Sci. U.S.A.* 111:6531. 10.1073/pnas.1404652111 24803289 PMC4020074

[B32] Martin R. E. Goodyear B. G. Gati J. S. Menon R. S. (2001). Cerebral cortical representation of automatic and volitional swallowing in humans. *J. Neurophysiol.* 85 938–950. 10.1152/jn.2001.85.2.938 11160524

[B33] Martino R. McCulloch T. (2016). Therapeutic intervention in oropharyngeal dysphagia. *Nat. Rev. Gastroenterol. Hepatol.* 13 665–679. 10.1038/nrgastro.2016.127 27625188

[B34] Mazurek K. A. Rouse A. G. Schieber M. H. (2018). Mirror neuron populations represent sequences of behavioral epochs during both execution and observation. *J. Neurosci.* 38 4441–4455. 10.1523/jneurosci.3481-17.2018 29654188 PMC5932647

[B35] Ogawa S. Tank D. W. Menon R. Ellermann J. M. Kim S. G. Merkle H. (1992). Intrinsic signal changes accompanying sensory stimulation: functional brain mapping with magnetic resonance imaging. *Proc. Natl. Acad. Sci. U.S.A.* 89 5951–5955. 10.1073/pnas.89.13.5951 1631079 PMC402116

[B36] Pelosin E. Barella R. Bet C. Magioncalda E. Putzolu M. Di Biasio F. (2018). Effect of group-based rehabilitation combining action observation with physiotherapy on freezing of gait in Parkinson’s disease. *Neural Plast.* 2018:4897276. 10.1155/2018/4897276 29977280 PMC5994277

[B37] Pelosin E. Bove M. Ruggeri P. Avanzino L. Abbruzzese G. (2013). Reduction of bradykinesia of finger movements by a single session of action observation in parkinson disease. *Neurorehabil. Neural Repair* 27 552–560. 10.1177/1545968312471905 23392919

[B38] Rizzolatti G. Craighero L. (2004). The mirror-neuron system. *Annu. Rev. Neurosci.* 27 169–192. 10.1146/annurev.neuro.27.070203.144230 15217330

[B39] Rizzolatti G. Sinigaglia C. (2016). The mirror mechanism: a basic principle of brain function. *Nat. Rev. Neurosci.* 17 757–765. 10.1038/nrn.2016.135 27761004

[B40] Rommel N. Hamdy S. (2016). Oropharyngeal dysphagia: manifestations and diagnosis. *Nat. Rev. Gastroenterol. Hepatol.* 13 49–59. 10.1038/nrgastro.2015.199 26627547

[B41] Sale M. V. Mattingley J. B. (2013). Selective enhancement of motor cortical plasticity by observed mirror-matched actions. *Neuroimage* 74 30–36. 10.1016/j.neuroimage.2013.02.009 23416734

[B42] Sale P. Franceschini M. (2012). Action observation and mirror neuron network: a tool for motor stroke rehabilitation. *Eur. J. Phys. Rehabil. Med.* 48 313–318. 22522432

[B43] Small S. L. Buccino G. Solodkin A. (2012). The mirror neuron system and treatment of stroke. *Dev. Psychobiol.* 54 293–310. 10.1002/dev.20504 22415917

[B44] Small S. L. Buccino G. Solodkin A. (2013). Brain repair after stroke—a novel neurological model. *Nat. Rev. Neurol.* 9 698–707. 10.1038/nrneurol.2013.222 24217509 PMC5549938

[B45] Soltysik D. A. Hyde J. S. (2006). Strategies for block-design fMRI experiments during task-related motion of structures of the oral cavity. *Neuroimage* 29 1260–1271. 10.1016/j.neuroimage.2005.08.063 16275020

[B46] Soros P. Inamoto Y. Martin R. E. (2009). Functional brain imaging of swallowing: an activation likelihood estimation meta-analysis. *Hum. Brain Mapp.* 30 2426–2439. 10.1002/hbm.20680 19107749 PMC6871071

[B47] Toogood J. A. Smith R. C. Stevens T. K. Gati J. S. Menon R. S. Theurer J. (2017). Swallowing preparation and execution: insights from a delayed-response functional magnetic resonance imaging (fMRI) study. *Dysphagia* 32 526–541. 10.1007/s00455-017-9794-2 28361202

[B48] Tramacere A. Pievani T. Ferrari P. F. (2017). Mirror neurons in the tree of life: mosaic evolution, plasticity and exaptation of sensorimotor matching responses. *Biol. Rev.* 92 1819–1841. 10.1111/brv.12310 27862868 PMC5433930

[B49] Ugurbil K. (2016). What is feasible with imaging human brain function and connectivity using functional magnetic resonance imaging. *Philos. Trans. R. Soc. Lond. B Biol. Sci.* 371:20150361. 10.1098/rstb.2015.0361 27574313 PMC5003861

[B50] Ushioda T. Watanabe Y. Sanjo Y. Yamane G.-Y. Abe S. Tsuji Y. (2012). Visual and auditory stimuli associated with swallowing activate mirror neurons: a magnetoencephalography study. *Dysphagia* 27 504–513. 10.1007/s00455-012-9399-8 22395851

[B51] Vesia M. Pellicciari R. Cash R. F. H. Isayama R. Kunaratnam N. Jegatheeswaran G. (2019). Learning from goal and action based observations differentially modulates functional motor cortical plasticity. *Neuroscience* 404 387–395. 10.1016/j.neuroscience.2019.02.019 30797894

[B52] Zhang J. J. Q. Fong K. N. K. Welage N. Liu K. P. Y. (2018). The activation of the mirror neuron system during action observation and action execution with mirror visual feedback in stroke: a systematic review. *Neural Plast.* 2018:2321045. 10.1155/2018/2321045 29853839 PMC5941778

